# Analytical performances achieved by the ‘Aria II’ automatic system in an interlaboratory survey triiodothyronine and thyroxine assays

**DOI:** 10.1155/S1463924683000395

**Published:** 1983

**Authors:** A. Pilo, G. C. Zucchelli, M. A. Piro, M. R. Chiesa

**Affiliations:** CNR Institute of Clinical Physiology c/o Università di Pisa Via Savi 8 Pisa 1 56100 Italy


Journal of Automatic Chemistry, Volume 5, Number 3 (July–September 1983), pages 157–158

### Analytical performances achieved by the 'Aria II' automatic system in an inter-laboratory survey for triiodothyronine and thyroxine assays

**A. Pilo, G. C. Zucchelli, M. A. Piro and M. R. Chiesa**

*CNR Institute of Clinical Physiology, c/o Università di Pisa, Via Savi 8, I 56100 Pisa, Italy*

### Introduction

During the last decade radioimmunological techniques have become a very useful diagnostic tool and many assays are frequently performed in clinical laboratories; this widespread use has prompted numerous attempts to partially or completely automate radioimmunoassay (RIA). Recently, a fully automatic system (the Aria II*), developed by Becton-Dickinson [1], has become widely used in laboratories which assay triiodothyronine ($T_3$) and thyroxine ($T_4$).

Automatic RIA systems must be evaluated in terms of their practicability (speed, cost and technical skill requirements) and also, and most importantly, in terms of their reliability (accuracy and precision). Results produced in a single laboratory have been used to evaluate the Aria II system [2 and 3]; data gathered in inter-laboratory surveys can also provide a useful means of comparing the performances of different analytical systems [4 and 5]. This paper describes an inter-laboratory approach—the accuracy and precision of the Aria II system has been estimated using data collected during a national external quality control survey (EQCS) for $T_3$ and $T_4$, which has been operated by the authors since January 1980.†

### Materials and methods

### *Main EQCS data*

Details of the EQCS organization and data processing have been reported elsewhere [6]. The present analysis is based on the results of 63 samples sent in 14 monthly despatches during the period April 1980 to November 1981; 31 of these samples, prepared from eight pools, were sent as hidden replicates (three to five replicates from each pool) to the participants in order to estimate their precision. The number of laboratories participating in the EQCS ranged from 39 (April 1980) to 148 (November 1981); 17 laboratories were using the Aria II system during the period November 1980 to November 1981, only one laboratory was using it before November 1980.

### *Data analysis*

The accuracy of the Aria II system was evaluated comparing the results obtained from laboratories using the automatic system with the consensus mean of the respective EQCS samples (taken as reference values). The precision of the Aria II was estimated from the results of hidden replicates.

*The Aria II is manufactured by Becton-Dickinson Immunodiagnostics, 180 West 2950 South, Salt Lake City, Utah 84115, USA.
† The external quality control survey is supported by the CNR Special Programme of Laboratory Medicine.

A CV was computed from the results of samples prepared from the same pool and a mean CV was obtained pooling the CVs of all the pools considered.

### *Recovery tests: preparation of samples and results*

The validity of the consensus mean as a reference value was checked by nine recovery experiments. These recovery tests were carried out by sending to participants either spiked and charcoal stripped sera. The 'free' serum was prepared by recycling for 24 h 100 ml serum on a 1·5 × 50 cm column filled with charcoal spherules (made by BAC [grade MU-LLI], Taiyo Kahen Co. Ltd, Tokyo, Japan). The stripping efficiency was monitored by adding labelled $T_3$ and $T_4$ to serum; in two experiments 98 to

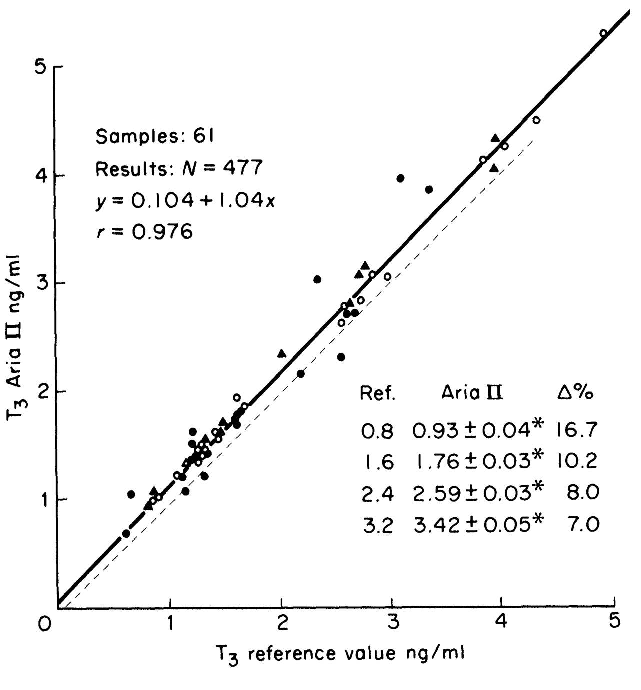

*Figure 1. Regression analysis of 477 $T_3$ results from laboratories using the Aria II system against the respective consensus means taken as reference values. The mean values found in each EQCS sample are indicated by different symbols depending on the number of laboratories using the Aria II (●: 1–5; ○: 6–10, ▲: 11–17); regression and identity are depicted as full and dashed lines respectively.*

*The inset table reports the mean readings by the Aria II system (computed from the regression line) corresponding to four assigned $T_3$ levels. Significance of the difference from the reference value ($p < 0{\cdot}01\%$; indicated by an asterisk) was estimated from the confidence limits of the regression line.*

*Table 1. Results of recovery tests from 'free serum' added to $T_3$ and $T_4$.*

| | $T_3$ | | | $T_4$ | | |
|---|---|---|---|---|---|---|
| Sample | $T_3$ concentration in added samples (ng/ml) | Mean of Aria II results (ng/ml) | Recovery % | $T_4$ concentration in added samples (μg/100 ml) | Mean of Aria II results (μg/100 ml) | Recovery % |
| C057 | 2·60 | 2·81 | 108·0 | 11·24 | 11·50 | 102·3 |
| C059 | 3·90 | 4·08 | 104·6 | 16·86 | 16·54 | 98·1 |
| C060 | 1·30 | 1·52 | 116·9 | 5·62 | 5·93 | 105·5 |

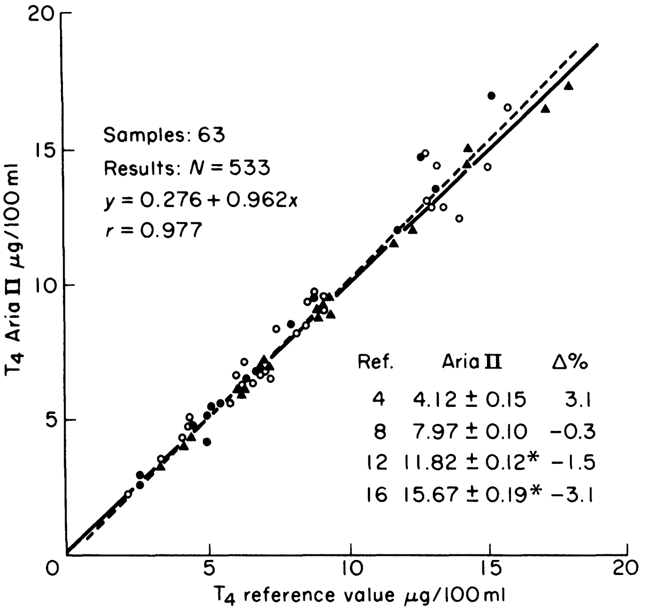

*Figure 2. See figure 1.*

99% of $T_3$ and 95 to 96% of $T_4$ could be removed. Stripped and spiked sera were added to $T_3$ and $T_4$ samples (purchased from Henning, Berlin, FR Germany) and measured by spectrophotometry. The results of the recovery experiments were 101·3% ± 4·3 SD and 101·8% ± 5·2 SD for $T_3$ and $T_4$ respectively; these findings strongly support the use of the consensus mean as reference value.

### Results and discussion

Figures 1 and 2 compare all the results obtained using the Aria II with the consensus means of the EQCS samples for $T_3$ and $T_4$ respectively. It can be seen that $T_3$ measurements produced by the automatic system are affected by a significant positive bias, which ranges from 17% in the low concentration values to 7% in the high values. As far as the $T_4$ assay is concerned, the Aria II results appear to be slightly biased (from 3% to −3% in the low and in the high $T_4$ values respectively). However, these small biases, even when statistically significant as mean values, are of low analytical relevance. The Aria II accuracy has been also directly evaluated from recovery tests; table 1 reports the mean results obtained by laboratories using the Aria II system in added samples, together with the spectrophotometrically measured concentrations of $T_3$ and $T_4$. These latter findings confirm that the automatic system overestimates $T_3$ concentrations, but that it assays $T_4$ without a significant bias.

*Table 2. Imprecision of the Aria II system and other methods.*

| Kit/method | Imprecision, $T_3$ | CV% $T_4$ |
|---|---|---|
| Ames, $T_3$, Seralute column separation $T_4$, Tetralute column separation-CPBA | 17·4 | 12·3 |
| Becton-Dickinson Aria II system | 12·4 | 9·2 |
| Biodata, PEG separation | 11·9 | 12·9 |
| Byk-Mallinckrodt, Spac. coated tubes | 17·3 | 12·7 |
| Clinical assays, coated tubes | 15·2 | 10·6 |
| Diagn. Prod. Corp., Double antibody with PEG separation | 15·3 | 11·3 |
| Lepetit, Liso-phase, column separation | 15·5 | 13·1 |

The pooled CVs computed from all hidden replicates are reported for the kit methods used by a substantial proportion of participating laboratories (there were at least 150 results for each 'kit' method).

The mean imprecision of the Aria II system achieved in the EQCS pools sent as hidden replicates during the whole period is shown in table 2; for comparison the table also shows the mean imprecision achieved by the six 'kit' methods more frequently used in the EQCS (by six to 20 participants). From these findings it is evident that the Aria II system attains a between-laboratory, between-batch precision which is similar, or better, than that obtained by the more precise method used in the EQCS. This good precision could, however, be expected if one considers the better standardization of an automatic analytical system compared with manual ones.

### References

1. Reese, M. G. and Johnson, L. R., *Clinical Chemistry*, **24** (1978), 342.
2. Chen, I. W., Maxon, H. R., Heninger, L. A., Ellis, K. S. and Valle, C. P., *Journal of Nuclear Medicine*, **21** (1980), 1162.
3. Pay, G. D. and Reese, M. R., *Journal of Solid-Phase Biochemistry*, **4** (1979), 15.
4. Wood, W. G., Bauer, M. and Horn, K. *et al.*, *Journal of Clinical Chemistry and Clinical Biochemistry*, **18** (1980), 511.
5. *Basic Ligand Assay Survey* (College of American Pathologists, Skokie, Illinois, quarterly report).
6. Pilo, A., Zucchelli, G. C. and Piro, M. A., *Journal of Nuclear Medicine and Allied Sciences*, **25** (1981), 107.

### BOOK REVIEWS

The Editor is pleased to receive new books for review in the Journal. Volumes on any aspect of chemical instrumentation are appropriate, including management economics and the general philosophy of automation.

Publishers should send review copies, or brochures, to:
*Dr Peter Stockwell, c/o Taylor & Francis Ltd, 4 John Street, London WC1N 2ET.*

